# Mitral valve repair with the semi-rigid Memo 4D annuloplasty ring: early clinical and echocardiographic outcomes from the MANTRA study

**DOI:** 10.1093/icvts/ivae208

**Published:** 2024-12-12

**Authors:** Omer Dzemali, Hector Rodriguez Cetina Biefer, Marco Di Eusanio, Olivier Fabre, Giovanni Troise, Nikolaos Bonaros, Francesco Grimaldi, Yeong-Hoon Choi, Giuseppe Santarpino, Cristian Baeza, Francesco Pollari, Bertrand Marcheix, Davide Pacini, Vincenzo Argano, Max Baghai, Moninder Bhabra, Enzo Mazzaro, Luigi Badano, Joerg Kempfert

**Affiliations:** Clinic for Cardiac Surgery, University Hospital of Zurich, Zurich, Switzerland; Clinic for Cardiac Surgery, City Hospital of Zurich—Triemli, Zurich, Switzerland; Clinic for Cardiac Surgery, University Hospital of Zurich, Zurich, Switzerland; Clinic for Cardiac Surgery, City Hospital of Zurich—Triemli, Zurich, Switzerland; Department for Cardiac Surgery, Ospedali Riuniti Ancona, Ancona, Italy; Department for Cardiac Surgery, Lens Hospital and Bois Bernard Private Hospital, Lens, France; Cardiac Surgery Unit, Poliambulanza Foundation Hospital, Brescia, Italy; Department of Cardiac Surgery, Innsbruck Medical University, Innsbruck, Austria; Department of Cardiac Surgery, IRCCS Policlinico San Donato, Milan, Italy; Department of Cardiac Surgery, Kerckhoff Klinik, Bad Nauheim, Germany; Department of Cardiac Surgery, Città di Lecce Hospital, Lecce, Italy; Department of Cardiac Surgery, University Hospitals Cleveland Medical Center, Cleveland, OH, USA; Department of Cardiac and Vascular Surgery, Klinikum Nürnberg-Paracelsus Medical University, Nuremberg, Germany; Department of Cardiac Surgery, CHU Toulouse Rangueil University Hospital, Toulouse, France; Unit of Cardiac Surgery, Policlinico S.Orsola-Malpighi, Bologna, Italy; The Cardiac Care Group, Policlinico Paolo Giaccone, Palermo, Italy; Cardiac Surgery, King’s College Hospital, London, UK; Division of Cardiac Surgery, Queen Elizabeth Medical Centre, Birmingham, UK; Az. Ospedaliero-Universitaria “Ospedali Riuniti” di Trieste, Trieste, Italy; Department of Cardiology, Istituto Auxologico Italiano, IRCCS, Milano, Italy; Department of Medicine and Surgery, University of Milano-Bicocca, Milan, Italy; Klinik für Herz-, Thorax- und Gefäßchirurgie Deutsches Herzzentrum der Charité, Berlin, Germany

**Keywords:** Mitral regurgitation, Surgical mitral valve repair, Annuloplasty semi-rigid ring, Memo 4D, Outcomes

## Abstract

**OBJECTIVES:**

Memo 4D is a semi-rigid ring with an exclusive saddle shape and progressive increased anteroposterior diameter. This preliminary analysis reports 30-day clinical and haemodynamic outcomes of the MANTRA Memo 4D sub-study.

**METHODS:**

MANTRA is an ‘umbrella’ prospective, multicentre, worldwide post-market study to collect real-life safety and performance data on the Corcym devices. Clinical and echocardiographic outcomes were gathered preoperatively, at discharge and each follow-up. KCCQ-12 questionnaires were collected preoperatively and at 30 days. Echocardiographic studies were performed per a predefined protocol and assessed by an independent core laboratory.

**RESULTS:**

In total, 166 patients (52, 31.3% female, mean age 60.7 ± 11.4 years) underwent mitral valve repair with Memo 4D in 17 international institutions between July 2021 and June 2023 (enrolment is still ongoing). Primary was the most common aetiology (157, 94.6%), of which 33 cases of Barlow’s disease (19.9%); secondary mitral regurgitation was present in six cases (3.6%). Thirty-day mortality was 0.6% (1). One stroke event (0.6%), one acute kidney failure (0.6%), one myocardial infarction (0.6%) and two reoperations within 30 days were reported. Surgery marked improvement in the patient’s NYHA class associated with a significant increase in KCCQ-12 summary score, from 69.1 (SD = 23.7) preoperatively to 83.9 (SD = 15.7) at 30 days. End-diastolic left ventricular diameters decreased from 55.19 (SD = 7.10) preoperatively to 52.70 (SD = 3.76) mm at 30 days, and left atrial volume decreased from 125.79 (SD = 46.33) preoperatively to 91.51 (SD = 37.20) ml at 30 days. Mitral regurgitation significantly reduced after the operation and up to 30-day follow-up.

**CONCLUSIONS:**

Mitral valve repair with Memo 4D is associated with good clinical and haemodynamic outcomes in the early period.

MANTRA ClinicalTrials.gov number NCT05002543.

## Graphical Abstract

**Figure:**
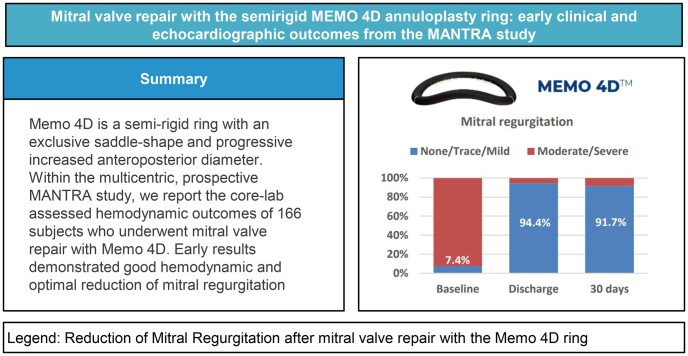

## INTRODUCTION

Surgical mitral valve repair (MVr) with an annuloplasty ring is effective, and it is considered the gold standard strategy to correct degenerative mitral valve regurgitation [1]. Annuloplasty allows re-shaping of the mitral annulus, reduces mitral regurgitation (MR) and restores proper leaflet coaptation and valve function [2]. Annuloplasty devices support the repaired leaflets and help prevent further annulus dilation [2]. Annular dilation and loss of its three-dimensional shape appear to be essential steps in the development of MR. Accordingly, restoring and preserving the normal annular geometry is critical for a successful repair [3]. Mitral annuloplasty rings can be classified as complete rings and partial bands. The original annuloplasty devices were rigid rings or bands. However, annuloplasty ring design has moved progressively towards more physiologically shaped and less rigid rings and bands. Mitral annuloplasty rings and bands are available in rigid, semi-rigid, and flexible formats.

Compared to other devices, complete semi-rigid rings quickly adapt to the shape of the native annulus [2, 4]. Indeed, the semi-rigid rings are mobile and can adjust to the saddle shape of the mitral valve during the cardiac cycle [4, 5] and remodel the mitral annulus without compromising its physiological movements [5].

The Memo annuloplasty ring family (Memo 3D, Memo 3D RECHORD, and Memo 4D, Corcym S.r.l, Saluggia, Italy) is composed of semi-rigid rings intended to accommodate the natural saddle shape of the mitral annulus and to support a comprehensive range of surgical techniques. Their semi-rigid nitinol core is designed to support both primary and secondary MR repair [3, 6–8]. Clinical data have demonstrated that Memo 3D could accommodate the physiological saddle shape of the mitral annulus throughout the cardiac cycle upon implantation [5, 9, 10].

Memo 4D is an evolution of Memo 3D presenting additional larger ring sizes (40 and 42 mm), increased anteroposterior dimension, and an out-of-plane saddle shape on the ring anterior portion, introduced for sizes 34–42 mm. These modifications are intended/designed to support the MVr in enlarged annuli that have lost their contractility and saddle-shape configuration.

Within the CORCYM Mitral, Aortic aNd Tricuspid Post-maRket Study in a reAl-world Setting (MANTRA) study (NCT05002543) [11], the Memo 4D sub-study aims to collect safety and device performance data on the annuloplasty ring with a dedicated echocardiographic core laboratory (Department of Cardiology, Istituto Auxologico Italiano, IRCCS, Milan, Italy) analysis of the product characteristics linked to haemodynamics.

The analysis aims to report the first real-world early clinical and haemodynamic outcomes obtained from patients who underwent MVr with the Memo 4D annuloplasty ring within the MANTRA study.

## PATIENTS AND METHODS

### Ethical statement

This study was approved by the Ethics Committees/Institutional Review Board and/or health authorities according to local regulations. All patients enrolled in the study provided written informed consent before undergoing any clinical investigation-specific assessments and treatments. Approval numbers from institutional review boards or ethics committees can be found under Supplementary Material, S6.

### Study protocol

Details about the design of the MANTRA Study have been published [11]. Briefly, the MANTRA study is a prospective, global, post-marketing study. The goal is to collect data on the safety and performance of devices for the Corcym cardiac surgery portfolio to treat aortic, mitral, and tricuspid valve diseases. The study uses a master protocol that outlines the main standard parameters, whereas the specific questions are addressed in the different substudies for aortic, mitral/tricuspid and Memo 4D devices. Primary and secondary end-points of the Memo 4D sub-study are reported in Supplementary Material, Appendix S2. The development of the study concept and the data collection forms have been extensively supported by a steering committee of expert cardiac surgeons and cardiologists.

The study allows for inclusion of isolated and combined procedures with multiple device implantation (sponsor or non-sponsor devices), with the possibility to follow safety and performance information on all the Corcym devices implanted and to collect data on the interaction of multiple valve treatments. All end-point definitions were adapted from the more recent guidelines for heart valve procedures [12, 13]. Haemodynamic and structural performance of the devices were collected preoperatively, intra-operatively, discharge, at 30 days (+14 days), at 12 months after implant and each subsequent follow-up using echocardiography as per the recommendations for the imaging assessment of prosthetic heart valves [14]. Additionally, procedure and hospitalization information were collected, including Enhanced Recovery After Surgery (ERAS) in sites using these protocols, and patient outcome measures such as New York Heart Association (NYHA) classification and quality-of-life questionnaires (Supplementary Material, Appendix S3).

### Study population

This analysis focused on the Memo 4D sub-study and on subjects diagnosed with mitral valve disease who were considered suitable to undergo surgical MVr with a Corcym Memo 4D annuloplasty ring.

### Study device

The Memo 4D device is a semi-rigid mitral annuloplasty ring made with a nickel–titanium alloy inner core enclosed in a sewing ring of silicone and knitted polyester fabric and coated with carbofilm.

The ring exhibits flexibility to accommodate the three-dimensional annulus motion.

Flexibility is maximal in the posterior curved portion and decreases gradually towards the anterior part to support remodelling. To facilitate the sizing of artificial chords, the ring has a series of removable loops made with a yellow surgical monofilament thread (‘RECHORD System’, RCS) in the posterior curve that supports sizing at annular plane level.

The Memo 4D annuloplasty ring is a variant of the Memo 3D device, introducing two larger ring sizes (40- and 42 mm) and an increased filler thickness in the outer portion of the ring. Sizes 34–42 mm have a different ring height/width proportion (i.e. increased anteroposterior distance) concerning the Memo 3D and an out-of-plane saddle on the ring anterior portion.

### Study procedures

The schedule follows standard-of-care practices and may vary across sites.

Clinical and echocardiographic assessments were obtained preoperatively, at implant, at hospital discharge, and at 30 days. Annual evaluations are planned until the 10-year follow-up and will be completed for all available patients. All patients have undergone a clinical assessment (NYHA class) and complete quality-of-life questionnaire (Kansas City Cardiomyopathy Questionnaire [KCCQ]-12 [15] at baseline, 30-day, and the 1-year follow-up). Haemodynamic data and structural performance data on the Memo 4D have been based on quantitative analysis performed by an independent echo core laboratory blinded to clinical data. Details on echocardiographic examination, as described in the echocardiographic protocol, are summarized in the Supplementary Material, Appendix S4. Considering the requirement of the study of having proper 3D images obtained from echocardiographic test, sites for the Memo 4D sub-study have been selected based on specific criteria, such as availability of dedicated echocardiographers and 3D echocardiography machine.

The study data are collected using a web-based electronic data capture system (Merative Clinical Development, Ann Arbor, MI, USA). The data are continuously reviewed for omissions, errors and values requiring further clarification via computerized and manual procedures. Serious adverse events, medical history and medications accompanying them are encoded using standard dictionaries. The enrolment is still ongoing.

### Statistical analysis

Variables are described as mean (SD) or median (interquartile range) for continuous variables and as number (%) for categorical variables. Outcomes are reported as descriptive statistics. The proportions of early adverse events were calculated as the total number of events divided by the total number of patients. Changes in KCCQ scores from baseline will be evaluated at 30 days using paired *t*-tests; however, in the present interim analysis, due to the incompleteness of the datasets, formal statistical comparison between preoperative and postoperative values was not done. No formal missing data analysis or imputation was performed. Missing data can be considered as ‘missing at random’, due to the implementation of strong edit checks and monitoring of the data. The statistical analyses were performed using SAS (Release 9.4, by SAS Institute Inc., Cary, NC, USA). Only up data up to 30-day follow-up data are presented in this manuscript.

## RESULTS

### Patient and operative data

Between July 2021 and June 2023, 166 patients underwent MVr with the Memo 4D annuloplasty ring in 17 institutions. All subjects were successfully implanted at the first attempt, except in two cases. One attempt failed because of mis-sizing, and the correct Memo 4D ring size was successfully implanted during the same procedure. In the second case, MVr was converted to mitral valve replacement because of persistent MR observed intra-operatively after annuloplasty. The characteristics of the study population are detailed in Table 1. Most of the study population were men (52, 31.3% female patients); the overall mean age was 60.7 (SD = 11.4) years, and their surgical risk was relatively low with a mean EuroSCORE II of 1.5 (SD = 1.4) and a mean the Society of Thoracic Surgeons (STS) score of 1.1 (SD = 1.1). Most patients (121, 73.9%) were in NYHA class II or III. The aetiology of the mitral valve disease was primary in most of the cases (157, 94.6%), of which 33 cases of Barlow’s disease (19.9%), and secondary in six cases (3.6%). One hundred forty-eight patients (89.2%) were classified as Carpentier type II (Table 1).

**Table 1:** Patient baseline characteristics

Parameters	Overall population (*N* = 166)
Age (years), mean (SD)	60.7 (11.4)
Female, *N* (%)	52 (31.3)
BSA (m^2^), mean (SD)	1.9 (0.2)
EuroSCORE II (%), mean (SD)	1.5 (1.4)
STS score (%), mean (SD)	1.1 (1.1)
NYHA class, *N* (%)	
Class I	42 (25.6)
Class II	84 (51.2)
Class III	37 (22.6)
Class IV	1 (0.6)
Not available	2
Comorbidities, *N* (%)	
Hypertension	65 (39.2)
Arrhythmia	42 (25.3)
Dyslipidaemia	38 (22.9)
Tobacco user	34 (20.5)
CAD	18 (10.8)
Obesity	17 (10.2)
Previous surgery, *N* (%)	
AF treatment	4 (2.4)
Pacemaker/defibrillator implantation	4 (2.4)
PCI	2 (1.2)
MV disease, *N* (%)	
Degenerative	157 (94.6)
Barlow’s disease	33 (19.9)
Functional	6 (3.6)
Others^a^	3 (1.8)
Carpentier classification, *N* (%)	
Type I—normal leaflet motion	13 (7.8)
Type II—leaflet prolapse	148^b^ (89.2)
Type III—restricted leaflet motion	5 (3.0)

a Two unknown, and one traumatic.

b One hundred and thirty-two posterior leaflets.

AF: atrial fibrillation; CAD: coronary artery disease; MV: mitral valve; PCI: percutaneous coronary intervention.

The operative data are reported in Table 2. Ninety-four (56.6%) patients were operated on via a minimally invasive right-sided mini-thoracotomy: 41 of them (43.6%) under direct vision, 23 in fully endoscopic and 29 under video-assisted approaches. A posterior leaflet resection was required in 55 cases (33.1%) and a cleft closure in 36 patients (21.7%). Posterior chordae replacements were performed in 54 cases (32.5%), while anterior chordae replacements in 12 cases (7.2%).

**Table 2:** Operative data

Parameters	Overall population (*N* = 166)
Surgical approach, *N* (%)	
Median sternotomy	72 (43.4)
Mini-thoracotomy^a^	94 (56.6)
Direct visualization	41 (43.6)
Fully endoscopic	23 (24.5)
Video-assisted	29 (30.9)
Repair details, *N* (%)	
Posterior leaflet resection	55 (33.1)
Anterior leaflet resection	4 (2.4)
Cleft closure	36 (21.7)
Alfieri/edge-to-edge repair	18 (10.8)
Replacement posterior chordae	54 (32.5)
Replacement anterior chordae	12 (7.2)
Concomitant procedures,^b^*N* (%)	
AVR	3 (1.8)
TVr	30 (18.1)
CABG	11 (6.6)
Pulse generator implant	1 (0.6)
AF treatment	31 (18.7)
Atrial septal defect repair	18 (10.8)
Aortic root replacement	1 (0.6)
Other^c^	19 (11.4)
Cardio-pulmonary bypass time (min), mean (SD)	122.6 (48.9)
Cross-clamp time (min), mean (SD)	87.5 ± 35.1
Memo 4D ring sizes, *N* (%)	
28	1 (0.6)
30	6 (3.6)
32	20 (12.0)
34	22 (13.3)
36	38 (22.9)
38	44 (26.5)
40	26 (15.7)
42	9 (5.4)

a One not available (NA).

b One subject can have more than one concomitant procedure.

c LAA closure/ligation, AV plasty, ascending aorta replacement, tricuspid valve reconstruction.

AVR: aortic valve replacement; CABG: coronary artery bypass graft; TVr: tricuspid valve repair.

The additional MVr techniques performed in addition to annuloplasty are reported in Table 2. The concomitant procedures performed in this series were aortic valve replacement (AVR) in three patients, tricuspid valve repair (TVr) in 30 cases (18.1%), atrial fibrillation (AF) treatment in 31 cases (18.7%), atrial septal defect repair in 18 cases (10.8%), and coronary artery bypass graft (CABG) in 11 cases (6.6%).

The size distribution is shown in Table 2, with more than 20% of patients (35) implanted with Memo 4D sizes 40–42 mm.

The mean duration of cardiopulmonary bypass was 122.6 (SD = 48.9) min, and the mean aortic cross-clamp time was 87.5 (SD = 35.1) min.

### Safety and patient-reported outcomes

The postoperative safety outcomes are summarized in Table 3, with 30-day mortality of 0.6% (1). In the early period, one stroke event (0.6%), one acute kidney failure (0.6%), one myocardial infarction (0.6%), and six (3.6%) cases of atrioventricular block III were reported. Reoperation of the mitral valve within the first 30 days occurred in two patients (both for worsening of MR, procedure-related because of the concomitant posterior leaflet resection and cleft closure procedure), who underwent mitral valve replacement. One hundred forty-nine patients completed the 30-day follow-up visit.

**Table 3:** Safety outcomes

Events	Early incidence (≤30 days) (*N* = 166)
Death (MOF)	1 (0.6%)
Re-intervention	2 (1.2%)
Stroke	1 (0.6%)
Transient global amnesia	1 (0.6%)
Acute kidney failure	1 (0.6%)
Endocarditis	0 (0.0%)
AV-block III	6 (3.6%)
Myocardial infarction	1 (0.6%)

AV: atrioventricular; MOF: multi-organ failure.

**Figure 1:**
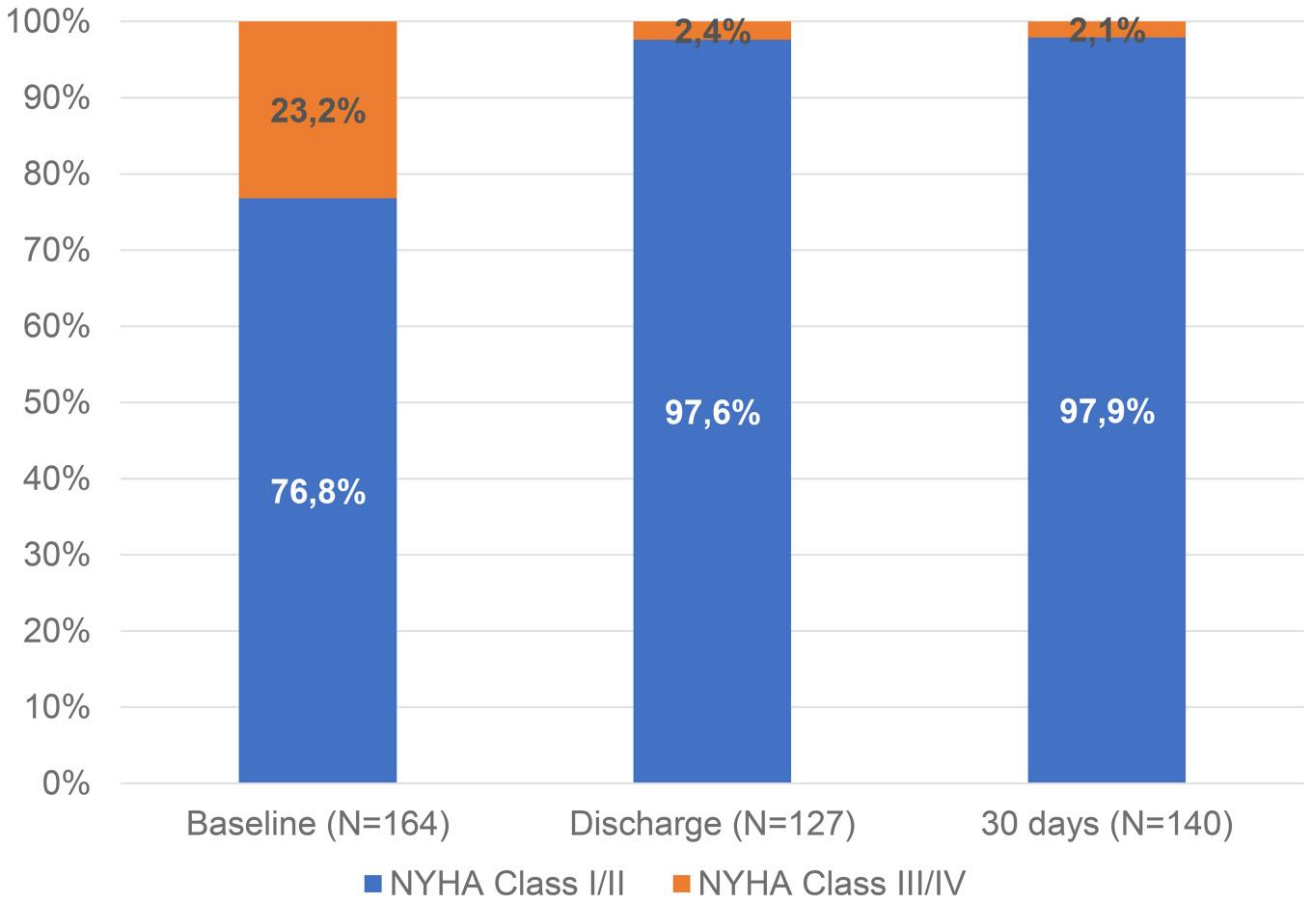
NYHA classification from preoperative to 30-day follow-up.

The assessment of functional status demonstrated a marked and stable improvement in NYHA class for most patients already at the 30-day follow-up visit. NYHA functional class I or II was recorded in 126/164 patients (76.8%) preoperatively versus 137/140 (97.9%) patients at the 30-day visit (Fig. 1).

**Figure 2:**
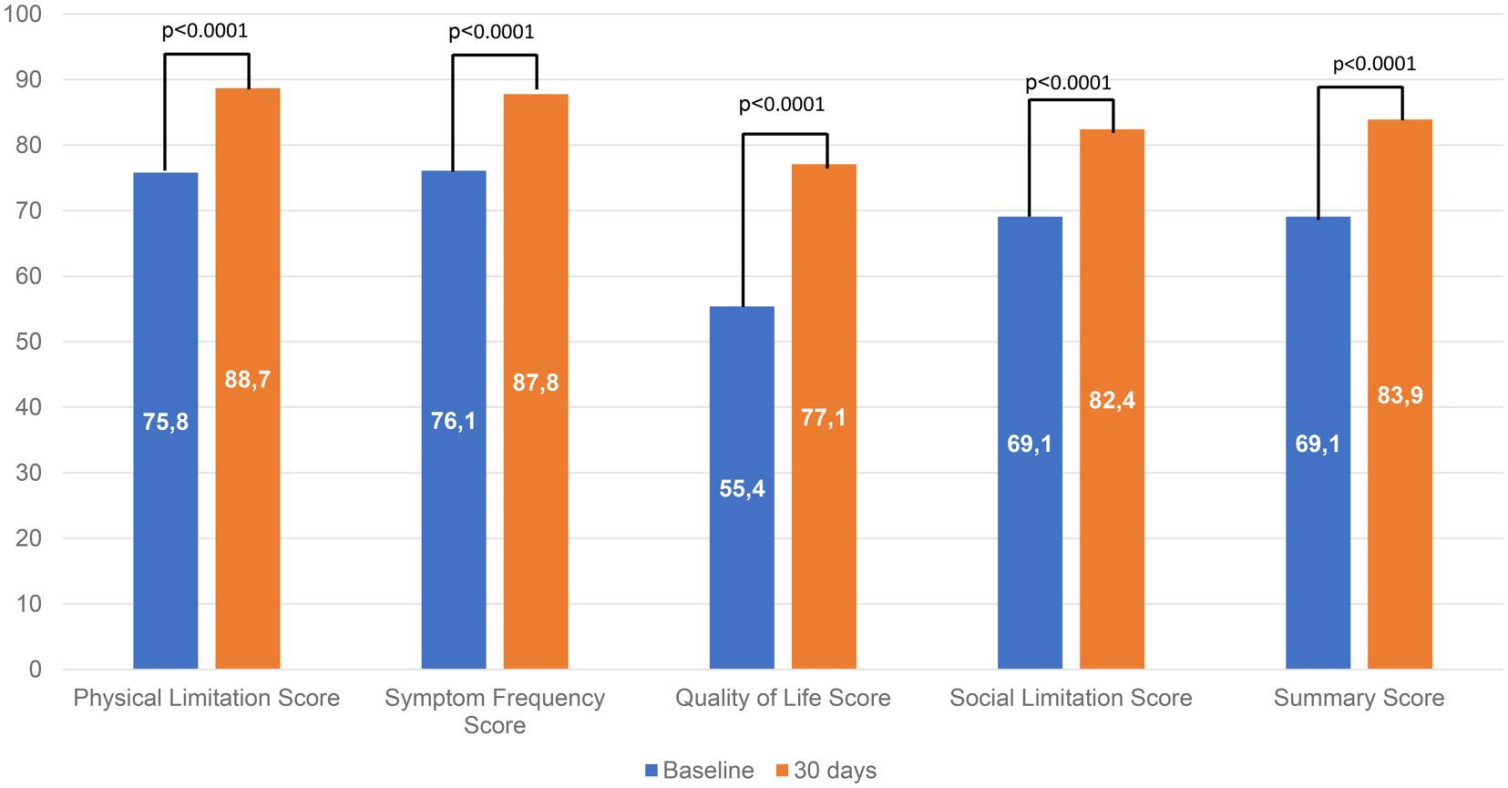
Mean KCCQ-12 scores at baseline and 30-day follow-up.

The KCCQ data were available for 165 patients (99.4%) preoperatively and 145 (97.3%) at 30 days. The KCCQ-12 summary score increased from 69.1 (SD = 23.7) before MVr to 83.9 (SD = 15.7) at 30 days (Fig. 2).

### Echocardiographic results

**Figure 3:**
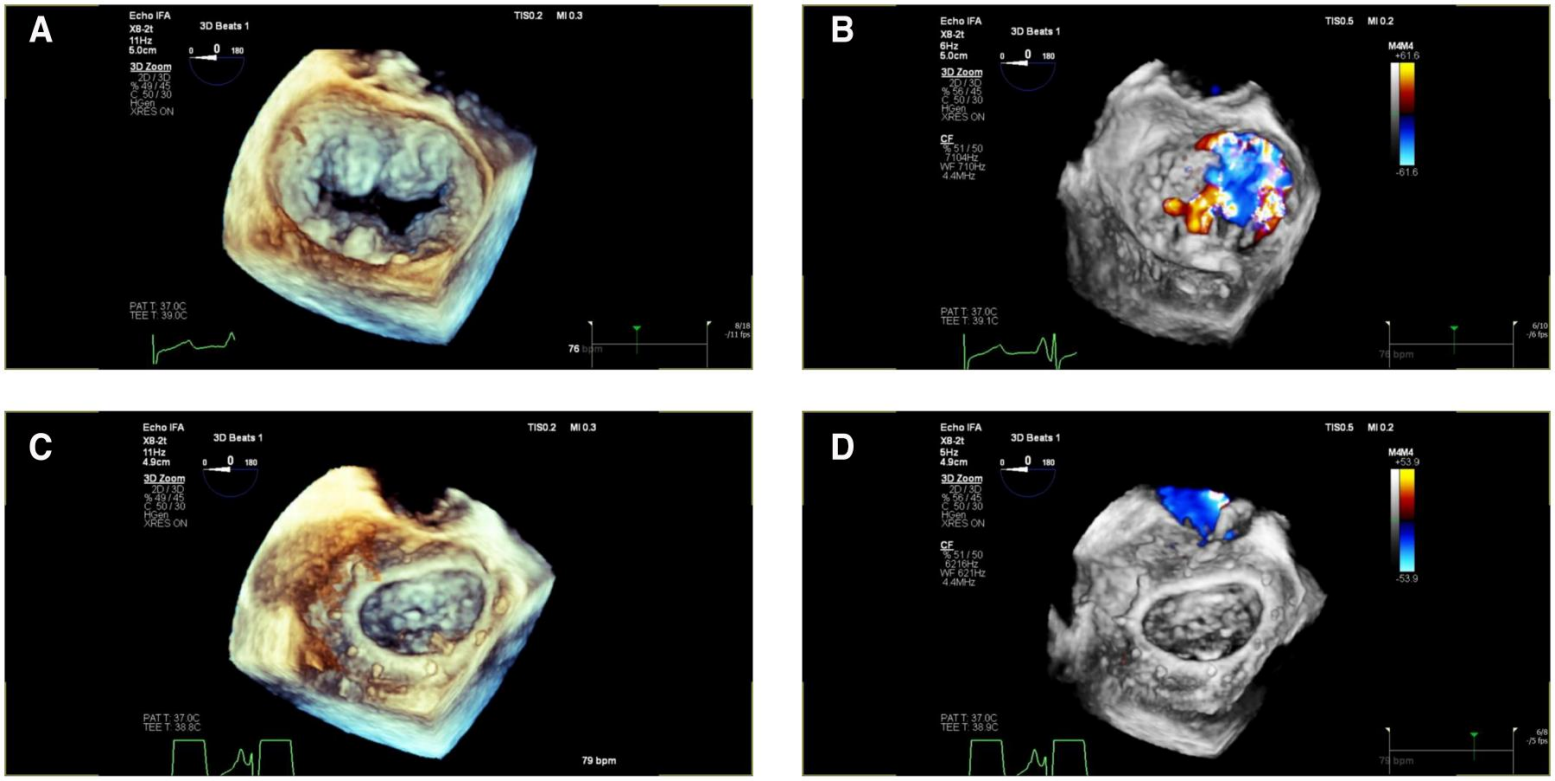
Three-dimensional transoesophageal echocardiography of the mitral valve in a patient with Barlow’s disease before (upper panels) and after mitral valve repair with Memo 4D annuloplasty ring (lower panels): en-face views of the mitral valve from the atrial perspective (**A**–**C**), colour Doppler (**B**–**D**).

Echocardiographic core-lab assessments are summarized in Table 4. Three-dimensional echocardiographic images of the mitral valve before and after MVr with the Memo 4D annuloplasty ring are shown in Fig. 3. Site-reported echocardiographic results are reported in Supplementary Material, Appendix S5.

**Table 4:** Echo-core-lab echocardiographic findings

	Baseline	Discharge	30 days
Left ventricular ejection fraction (%)			
*n*	90	111	101
Mean (SD)	65.08 (9.66)	54.26 (9.62)	54.28 (9.43)
Median	66.50	54.00	55.00
IQR	60.00; 73.00	49.00; 62.00	50.00; 61.00
Left ventricular end-diastolic diameter (mm)			
*n*	101	119	106
Mean (SD)	55.19 (7.10)	53.62 (41.28)	52.70 (32.76)
Median	56.00	50.00	50.00
IQR	50.00; 60.00	44.00; 56.00	45.00; 54.00
Left ventricular end-systolic diameter (mm)			
*n*	100	113	102
Mean (SD)	36.06 (9.34)	43.23 (48.29)	35.73 (7.68)
Median	36.00	37.00	35.00
IQR	29.00; 42.00	32.00; 43.00	30.00; 41.00
Left ventricular end-diastolic volume (ml)			
*n*	90	111	101
Mean (SD)	164.87 (52.60)	133.94 (44.10)	127.43 (38.49)
Median	160.50	128.00	122.00
IQR	120.00; 201.00	103.00; 162.00	104.00; 144.00
Left ventricular end-systolic volume (ml)			
*n*	90	111	101
Mean (SD)	57.92 (25.91)	63.14 (28.31)	59.14 (26.14)
Median	52.50	59.00	55.00
IQR	40.00; 68.00	40.00; 78.00	45.00; 67.00
Left atrial volume (ml)			
*n*	96	100	100
Mean (SD)	125.79 (46.33)	108.00 (35.65)	91.51 (37.20)
Median	114.00	105.50	86.50
IQR	91.00; 161.50	82.00; 129.50	70.00; 107.00
Mean mitral pressure gradient (mmHg)			
*n*		113	107
Mean (SD)		4.02 (1.81)	3.42 (1.42)
Median		4.00	3.00
IQR		3.00; 5.00	2.00; 4.00
Mitral valve area (cm^2^)			
*n*		57	92
Mean (SD)		2.29 (0.69)	2.41 (0.70)
Median		2.10	2.30
IQR		1.70; 2.70	1.90; 3.00
Systolic anterior motion, *N* (%)		*N* available = 63	*N* available = 46
Yes	NA	0 (0.0%)	0 (0.0%)
No	NA	63 (100.0%)	46 (100.0%)
Mitral regurgitation, *N* (%)	*N* available = 121	*N* available = 125	*N* available = 108
None	3 (2.5%)	70 (56.0%)	45 (41.7%)
Trace	1 (0.8%)	12 (9.6%)	14 (13.0%)
Mild	5 (4.1%)	36 (28.8%)	40 (37.0%)
Moderate	28 (23.1%)	5 (4.0%)	7 (6.5%)
Severe	84 (69.4%)	2 (1.6%)	2 (1.8%)

IQR: interquartile range; SD: standard deviation.

MVr with Memo 4D annuloplasty ring resulted in an immediate decrease of the left ventricular end-diastolic diameter [from 55.19 (SD = 7.10) mm preoperatively to 52.70 (SD = 32.76) mm at 30 days], a decrease in the left ventricular end-diastolic volumes [from 164.87 (SD = 52.59) ml preoperatively to 127.43 (SD = 38.48) ml at 30 days] and a reduction in left atrial volume [from 125.79 (SD = 46.33) ml preoperatively to 91.51 (SD = 37.20) ml at 30 days]. No cases of systolic anterior motion were reported. 30-day mean mitral gradient was 3.42 (SD = 1.42) mmHg.

MR decreased significantly after the operation and was stable at the 30-day follow-up. The echocardiographic examinations, including the results of the patients who required a reoperation, revealed no or mild MR in 99 of 108 available assessments (91.7%) at 30 days, moderate MR in seven patients (6.5%) and severe MR in two patients (1.8%).

## DISCUSSION

This study reported for the first time the clinical and echocardiographic results of the Memo 4D mitral ring in a prospective, multicentre, international real-world experience.

The data demonstrated that MVr with the Memo 4D improved the patient’s clinical status with a significant increase in quality of life and good early haemodynamic performance with optimal reduction of the severity of MR and preservation of the left ventricular function.

The Memo annuloplasty semi-rigid rings (Corcym S.r.l, Saluggia, Italy) have been commercially available for several years to support MVr in both primary and secondary MR [3, 6–8], showing that the devices are safe and effective when used alone or in combination with other techniques for MVr.

The design improvement of the Memo 4D ring addresses the need for larger ring sizes. It features a ring re-shaping from size 34 to 42 mm, including an increased anteroposterior dimension and an out-of-plane saddle shape on the ring anterior portion. The result of this preliminary study reflects that adding larger sizes in the Memo 4D ring met an actual surgical need since a 40–42 mm ring was required in more than 20% of the implanted patients.

In our series, we report low morbidity and mortality at 30 days and a consistent and significant improvement in the quality of life that was evident in the early period. The observed 30-day mortality of 0.6% is in line with outcomes of similar devices [16], demonstrating that, in modern cardiac surgery, elective MVr for primary MR is one of the safest procedures [17].

Of note, the excellent safety and patient-reported outcomes in this series were obtained with more than half of the patients treated in a minimally invasive approach, in line with the vision of reducing invasiveness, postoperative pain, complications and hospital stays [18, 19]. Although transcatheter technology is rapidly growing and represents a promising strategy for valve replacement [20], the surgical approach remains the best technique to repair a primary MR [1].

The effectiveness and the safety of the Memo 4D are also underlined by the improved functional status of the patients from preoperative to 30 days. Patients in NYHA class I or II increased from 126 preoperatively to 137 (of those with available data) at 30 days, showing similar outcomes as previously reported in another study with Memo 3D [8]. In addition, the KCCQ-12 summary score increased from 69.1 (SD = 23.7) before MVr to 83.9 (SD = 15.7) at 30 days, indicating a moderate to significant clinical improvement [15]. Finally, the echocardiography data analysed by the core laboratory confirm the effectiveness of MVr Memo 4D ring. There were no left ventricular outflow tract (LVOT) obstructions due to systolic anterior motion.

Furthermore, MR degree decreased significantly after the operation and was maintained at the 30-day follow-up, with a low incidence of reoperation due to recurrent moderate/severe insufficiency. These echocardiographic findings are in line with previously published experience with Memo 3D and similar devices [10, 16].

Recent clinical data have shown that Memo 3D was able to preserve the movement of the anterior and posterior portion of the mitral annulus, as well as changes in the annulus geometry throughout the cardiac cycle [5, 9, 10]. In this regard, to better understand the relationship between the saddle-shaped Memo 4D annuloplasty ring and the annular motion post-implant and through the follow-up, in the MANTRA study, once all enrolled patients will reach the 1-year visit, the 3D echocardiography datasets will be analysed by the echocardiographic core laboratory using advanced imaging technique, focusing on the analysis of restoration of the annulus saddle shape configuration and the dynamics of the mitral annulus.

### Study limitations

This study has the limitations of any observational study involving no adjudication of patient inclusion and adverse events. It is a prospective, non-randomized study, lacking a comparative arm. Furthermore, midterm and long-term echocardiographic characteristics are missing because most patients were included in the past year. Some examinations are still to be collected and read by the independent echocardiography core laboratory.

## CONCLUSION

In conclusion, the present study details the early preliminary results of patients undergoing MVr with the Memo 4D annuloplasty ring in a prospective, multicentre, international real-world experience, with the haemodynamic assessments performed by an independent core laboratory. The data demonstrated good clinical outcomes, moderate-to-large improvement of the patient’s quality of life at 30 days and good early haemodynamic performance with optimal reduction of the severity of MR and preservation of the left ventricular function.

Further clinical and echocardiographic evaluations with a larger patient cohort and a longer follow-up period are ongoing to confirm the results of this preliminary experience.

## Supplementary Material

ivae208_Supplementary_Data

## Data Availability

All relevant data are within the manuscript and its supporting information files.

## ABBREVIATIONS

ERAS: Enhanced recovery after surgery
KCCQ: Kansas City Cardiomyopathy Questionnaire
MR: Mitral regurgitation
MVr: Mitral valve repair
NYHA: New York Heart Association
RCS: RECHORD system
SD: Standard deviation
