# Supplementary material for: Mitral valve repair with the semi-rigid Memo 4D annuloplasty ring: early clinical and echocardiographic outcomes from the MANTRA study
Source: Interdiscip Cardiovasc Thorac Surg. 2024 Dec 12;40(1):ivae208. doi: 10.1093/icvts/ivae208 (PMC11700519; doi:10.1093/icvts/ivae208)
Supplement: ivae208_Supplementary_Data [file ivae208_supplementary_data.docx]

**Early Outcomes with MEMO 4D mitral ring results from the MANTRA study**

**SUPPLEMENTARY APPENDIX**

**Contents**

[S1_MANTRA MEMO 4D sub-study INVESTIGATORS AND CONTRIBUTORS 2](#_Toc174133845)

[S2_MANTRA MEMO 4D sub-study endpoints 6](#_Toc174133846)

[S3_MANTRA MEMO 4D sub-study schedule of assessments 11](#_Toc174133847)

[S4_MEMO 4D ECHOCARDIOGRAPHIC PROTOCOL 13](#_Toc174133848)

[S5_Table S5_ Site reported echocardiographic findings 17](#_Toc174133849)

[S6_Approval numbers from Institutional Review Boards or Ethic Committees 19](#_Toc174133850)

# S1_MANTRA MEMO 4D sub-study INVESTIGATORS AND CONTRIBUTORS

**MANTRA STEERING COMMITTEE**

- Bart Meuris, UZ Gasthuisberg Leuven, University Hospital, Leuven, Belgium
- Patrizio Lancellotti, University Hospital Liège, CHU Sart Tilman, Liège,Belgium
- Serdar Günaydın, Ankara City Hospital, Ankara, Turkey
- Jörg Kempfert, Klinik für Herz-, Thorax- und Gefäßchirurgie Deutsches Herzzentrum der Charité, Berlin, Germany
- Gabriel S. Aldea, University of Washington, Seattle, WA, USA

**MANTRA ECHOCARDIOGRAPHIC CORE-LAB**

Department of medicine and surgery, University Milano-Bicocca

Department of Cardiovascular, Neural and Metabolic Sciences, Istituto Auxologico Italiano, IRCCS

Principal Investigators

Luigi P. Badano, MD, PhD, FESC, FACC

Denisa Muraru, MD, PhD, FESC, FACC

**STUDY SITES, INVESTIGATORS AND KEY PERSONNEL**

| **Site** | **Personnel** |
| --- | --- |
| Stadtspital Triemli, Birmensdorferstrasse 497, 8063 Zurich, Switzerland | Principal Investigator  Omer Dzemali  Sub-Investigators  Hector Rodriguez, Philine Fleckenstein, Nestoras Papadopoulos  Study coordinators  Jessica Hohn |
| Ospedali Riuniti Ancona, Via Conca, 71-Torrette, 60126 Ancona, Italy | Principal Investigator  Marco Di Eusanio  Sub-Investigators  Olimpia Bifulco, Jacopo Alfonsi, Paolo Berretta, Carla Lofiego, Leonardo Brugiatelli, Fabio Vagnarelli |
| Fondazione Poliambulanza Istituto Ospedaliero, via Bissolati, 57, 25124 Brescia, Italy | Principal Investigator  Giovanni Troise  Sub-Investigators  Margherita Dalla Tomba, Emmanuel Villa, Antonio Messina |
| Centre Hospitalier de Lens, 99 Route de la Bassée, 62320 Lens, France | Principal Investigator  Olivier Fabre  Sub-Investigators  Ilir Hisy, Laurence Gautier |
| Kerckhoff Klinik, Benekestr. 2 – 8, 61231, Bad Nauheim, Germany | Principal Investigator  Yeong-Hoon Choi  Sub-Investigators  Olivier Liakopoulos, Silvana Hamati, Ayse Cetinkaya, Efstratios Charitos, Majd Ismail, Markus Schoenburg, Zoltan Szalay, Mohamed Zeriouh,  Study Coordinators  Rita Michel |
| Città di Lecce Hospital, Via Provinciale per Arnesano km 4, 73100 Lecce, Italy | Principal Investigator  Giuseppe Santarpino  Sub-Investigators  Anna Nicoletti, Corrado Fiore  Study Coordinators  Chiara Coppola |
| IRCCS Policlinico San Donato, Piazza Edmondo Malan, 2, 20097, Milano, Italy | Principal Investigator  Francesco Grimaldi  Sub-Investigators  Lorenzo Menicanti, Giacomo Bortolussi, Marianna Volpe, Elisa Gastino |
| Medical University of Innsbruck Christoph-Probst-Platz 1, Innrain 52 A – 6020, Innsbruck, Austria | Principal Investigator  Nikolaos Bonaros  Sub-Investigators  Daniel Hofer, Leo Polzl, Jacob Hirsch  Study coordinators  Monica Auer |
| University Hospitals Cleveland Medical Center, 11100 Euclid Ave, Cleveland, OH 44106, USA | Principal Investigator  Cristian Baeza  Sub-Investigators  Marc Pelletier, Yasir Abu-Omar, Pablo Ruda Vega, Gregory Rushing, Lina El Hajj  Study Coordinators  Sarah Mitchell, Emily Mullenax, GaMia Dix, Mallory Lane Vaughn, Stacey Mazzurco |
| Klinikum Nürnberg, Paracelsus Medical University, Breslauer Str. 201, 90471, Nuremberg, Germany | Principal Investigator  Francesco Pollari  Sub-Investigators  Theodor Fischlein, Erik Bagaev, Islam Batashev, Christina Bermel, Matthias Fittkau, Faig Guliyev, Philipp Bauernschubert  Study coordinators  Fatima Jiven-Jetzelsberger, Apolonija Kalisnik |
| Deutsches Herzzentrum der Charité, Augustenburger Platz 1, 13353 Berlin, Germany | Principal Investigator  Joerg Kempfert  Sub-Investigators  Nicolas Merke, Markus Kofler, Karel van Praet  Study Coordinators  Mirijam Gordes |
| CHU Toulouse_Rangueil University Hospital, avenue Jean Poulhes 1, 31050 Toulouse, France | Principal Investigator  Bertrand Marcheix  Sub-Investigators  Roxana Botea, Christophe Cron  Study Coordinators  Dylan Benane |
| Policlinico S.Orsola-Malpighi, Via Giuseppe Massarenti 9, 40138, bologna, Italy | Principal Investigator  Davide Pacini  Sub-Investigators  Gianluca Folesani, Riccardo Nania, Elena Biagini |
| Policlinico Paolo Giaccone, Via del Vespro 129, 90127 Palermo, Italy | Principal Investigator  Vincenzo Argano  Sub-Investigators  Sebastiano Castrovinci, Salvatore Torre, Enrico Amoncelli, Enza Tortorici, Salvatore Territo, Antonio Segreto |
| King’s College Hospital, Denmark Hill, SE5 9RS London, UK | Principal Investigator  Max Baghai  Sub-Investigators  Alexandros Papachristidis  Study Coordinators  Sarah Byrne, Abigail Knighton, Jonathan Breeze, Mariya Tomi, Hosanna Assefa-Kebede |
| Queen Elisabeth medical Centre, Mindelsohn Way, B15 2TH Birmingham, UK | Principal Investigator  Moninder Bhabra  Sub-Investigators  Ravi Hebballi, Amor Mia Alvior  Study Coordinators  Annette Nilson, Helen Botherton, Tabassuma Akramul, Kahmeelah Dowling |
| Az. Ospedaliero-Universitaria “Ospedali Riuniti” di Trieste, Via della Pietà 19, 34129 Trieste, Italy | Principal Investigator  Enzo Mazzaro  Sub-Investigators  Elisabetta Rauber, Ilaria Franzese, Angela Poletti |

**PROJECT MANAGEMENT (CORCYM S.r.l.)**

Elisa Cerutti, Sr Clinical Project Manager

Luca Foppoli, Manager, Clinical Operations

Silvia Dipinto, Clinical Project Manager

Nelly Rivera, Associate Project Manager

Michela Paroli, Associate Clinical Project Manager

Elona Mulai, Associate Clinical Project Manager

Mara Chiaro, Head Clinical, Quality and Regulatory Affairs

Sara Gaggianesi, Medical Affairs Director

Paola Morando, Principal Data Manager

Laura Chiara, Sr. Data Quality Specialist

**STATISTICAL and PROGRAMMING CONSULTANT**

Valos S.r.l, via Ceccardi 4/31, 16121, Genova, Italy

# S2_MANTRA MEMO 4D sub-study endpoints

**Primary endpoint**

Primary endpoint will be device success at 30 days (+14 days) adapted from the MVARC^[[1]](#footnote-2)^, defined as

- Procedural mortality or stroke; AND
- Proper placement and positioning of the device; AND
- Freedom from unplanned surgical or interventional procedures related to the device or access procedure; AND
- Continued intended safety and performance of the device, including:
  - Evidence of structural or functional failure
  - Specific device-related technical failure issues and complications
  - Reduction of MR to either optimal or acceptable levels without significant mitral stenosis (i.e., post-procedure EOA is ≥1.5 cm^2^ or a transmitral gradient <5 mmHg), and with no greater than mild MR core-lab assessed

**Secondary endpoints**

The secondary endpoints include:

**Safety and performance assessments at 30 days, 12 months and annually at each follow up after implant:**

- All-cause mortality
- Cardiac mortality
- Device related mortality
- Major Adverse Cardiovascular and Cerebrovascular Event (MACCE - composite endpoint of all cause death, myocardial infarction, stroke, and valve re-intervention)
- Device related Serious Adverse Events
- All cause re-hospitalization
- Re-hospitalization related to cardiovascular causes.
- Device related re-hospitalization.
- Technical success, defined as successful delivery, correct positioning and deployment of the first intended device intraoperatively.
- Hemodynamic and structural performance from site-reported echocardiography intra-operatively, discharge, at 30 days (+ 14 days), at 12 months after implant and at each subsequent follow-up, if available, including:
  - Mean Pressure Gradient (MPG)
  - Peak Pressure Gradient (PPG)
  - Left Ventricular End-Diastolic Volume (LVEDV)
  - Left Ventricular End-Systolic Volume (LVESV)
  - Left Ventricular End-Diastolic Diameter (LVEDd)
  - Left Ventricular End-Systolic Diameter (LVESd)
  - Left Ventricular Ejection Fraction (LVEF)
  - Incidence and degree of mitral regurgitation (None, Trace, Mild, Moderate, Severe)
  - Effective Regurgitant Orifice Area (EROA)
  - Left Ventricular Outflow Tract (LVOT) obstruction.
  - Left Ventricular Outflow Tract (LVOT) diameter.
  - Systolic Anterior Motion (SAM)
  - Incidence and degree of Tricuspid regurgitation (TR)
  - Estimated mean pulmonary artery pressure.
  - Left Atrial volume (LA volume)
  - Cardiac Output (CO)

collected as per the published recommendations for the imaging assessment of prosthetic heart valves^[[2]](#footnote-3)^

- Hemodynamic and structural performance assessed by echocardiographic core lab intra-operatively, discharge, at 30 days (+ 14 days), at 12 months, and at each subsequent follow-up, if available, including:
- Annular height commissure width ratio (AHCWR)
- Mean Pressure Gradient (MPG)
- Peak Pressure Gradient (PPG)
- Left Ventricular End-Diastolic Volume (LVEDV)
- Left Ventricular End-Systolic Volume (LVESV)
- Left Ventricular End-Diastolic Diameter (LVEDd)
- Left Ventricular End-Systolic Diameter (LVESd)
- Left Ventricular Ejection Fraction (LVEF)
- Antero posterior annulus diameter
- Commissure to commissure diameter, and circumference
- Left Ventricular Outflow Tract (LVOT) diameter.
- Systolic Anterior Motion (SAM)
- Annular Height
- Coaptation length
- Mean diastolic pressure gradient.
- Incidence and degree of Mitral regurgitation (MR) (None, Trace, Mild, Moderate, Severe)
- Effective Regurgitant Orifice Area (EROA)
- Effective Orifice Area (EOA)
- Incidence and degree of Tricuspid regurgitation (TR)
- Estimated mean pulmonary artery pressure.
- Left Atrial volume (LA volume)
- Cardiac Output (CO)
- Global left ventricular longitudinal strain

**Collection of procedural and hospitalization information** such as:

- Valve lesion type, pathology, and etiology
- Surgical Approach
- Ring size
- Use of RECHORD system
- Concomitant procedures
- Deficiencies related to the utilization of the devices and/or accessories.
- Operative room time
- Total procedure time
- Cardio-Pulmonary Bypass and Cross-clamp time
- Ventilation time
- Duration of Intensive Care Unit (ICU)
- Pain score assessment (Visual Analogue Scale)
- Number of blood transfusion(s)
- Anticoagulant Medication
- Duration of index hospitalization

**Patient outcome**:

- Quality of Life (Kansas City Cardiomyopathy Questionnaire - KCCQ) improvement from baseline up to 12 months
- Change in New York Heart Association (NYHA) functional class from baseline up to 12 months after implant and at each subsequent follow-up.

The improvement of the patient’s clinical status is assessed through:

- the NYHA functional class, which is expected to improve of ≥1 functional class.
- the patients’ Quality of Life (QoL) (Kansas City Cardiomyopathy Questionnaire), which is expected to improve of ≥10 and that according to Spertus et al, is considered a moderate-to-large clinical change^[[3]](#footnote-4)^.

The improvement of the valve hemodynamics is demonstrated through the reduction of MR from baseline. The post-procedure MR reduction is considered optimal when is reduced to trace or absent; it is considered acceptable when it is reduced by at least of 1 class or grade from baseline and to no more than moderate in severity^1^.

Additional analysis may be conducted as needed and will be specified in the statistical analysis plan.

Although this is a post-market study, the ISO 5910 guidance was taken into consideration for study sample size and duration. The ISO 5910 recommends a minimum of 150 subjects with 400 patient-years follow-up for new heart valve repair devices. The planned sample size of this post-market study is 200 subjects with following-up up to 10 years, which meets the sample size, and it is expected to also meet the follow-up duration requirement in the pre-market setting. This sample size allows for an adequate monitoring of the safety and performance of the Corcym Memo 4D devices in a real-world setting.

The sample size calculation is performed for the newly introduced saddle-shaped sizes of Memo 4D ring meant to address the need for larger ring sizes with an optimized shape to treat highly degenerated mitral valves. The endpoint of the sample size calculation is the restoration of the AHCWR during end diastole and end systole of the cardiac cycle at 12 months (± 30 days) after the implantation, compared to baseline. An improvement in AHCWR at post-implant time points compared to baseline indicates that the valve functions properly.

The sample size calculation is performed for saddle-shaped sizes Memo 4D mitral annuloplasty rings only, using the *t* test for paired means (i.e., before and after device implant) with the following parameters:

- 90% power
- 5% significance level, one-sided
- Standard deviation=8
- μ_d_=5%, assuming a baseline AHCWR value of 15% and a post-operative value at 12 months of 20%.

The AHCWR values are reported to be in the range of 20-30% when a mitral valve functions properly and in the range of 10-17% for diseased valve^[[4]](#footnote-5),^ ^[[5]](#footnote-6),^ ^[[6]](#footnote-7),^ ^[[7]](#footnote-8),^ ^[[8]](#footnote-9)^.

Ryomoto et al, 2014 is reporting the evaluation of mitral annular structure and dynamics of three investigated rings, two featuring a saddle-shape configuration (Physio II, Rigid Saddle Shape) and one semirigid ring (Memo 3D). The preoperative AHCWR in diseased patients was reported to be in the range of 13-15%, while the normal control value was around 20%. The two rings featuring a saddle-shape configuration led to increase of AHCWR of 5% in the immediate post-operative period maintained also in the mid-term follow up^5^.

As highlighted, MEMO 4D with saddle-shape configuration in larger sizes, allow its use in enlarged annuli, which lose contractility and the ability to saddle-shape in systole, where AHCWR is expected to be in the range of 10-15%. The expected increase of 5% in the AHCWR allowing to reach values comparable to healthy subjects can restore the three-dimensional leaflet curvature and reduce the leaflet stress improving mitral valve functions ^8^ and therefore leading to a meaningful clinical improvement.

The sample size calculated by using the above parameters is 41 for Memo 4D featuring saddle-shape configuration (34-42 mm). The total study sample is 200 and it is projected that at least 60 subjects will be treated with Memo 4D saddle-shape configurated rings, therefore meeting the sample size requirement for the hypothesis testing.

# S3_MANTRA MEMO 4D sub-study schedule of assessments

| **Item** |  |  |  |  |  |  |
| --- | --- | --- | --- | --- | --- | --- |
|  | **Screening/ Baseline** | **Procedure** | **Hospital Discharge** | **30D (+ 14D)** | **12M**  **(+/- 30D)** | **Annual visits (to ten years)^3^** |
| Type of Visit | Office/  Clinic | Office/  Clinic | Office/Clinic | Office/  Clinic | Office/  Clinic | Office/  Clinic  Phone  Referring physician |
| Informed Consent | X |  |  |  |  |  |
| Eligibility Criteria | X |  |  |  |  |  |
| Demographics | X |  |  |  |  |  |
| Medical History and risk factors | X |  |  |  |  |  |
| Clinical assessment | X |  |  |  |  |  |
| Vital Signs | X |  | X | X | X | X |
| Medications^1^ | X |  | X | X | X | X |
| Blood Test^2^ | X^,^ |  | X | X | X | X |
| Procedural details of implantation and concomitant procedures |  | X |  |  |  |  |
| ICU Management |  |  | X |  |  |  |
| Hospitalization data |  |  | X |  |  |  |
| Pain score assessment (VAS)^4^ |  |  | X^4^ | X^4^ |  |  |
| Echocardiography^5^ | X | X | X | X | X | X |
| *CT^6^* | *X* | *X* | *X* | *X* | *X* | *X* |
| Electrocardiogram | X |  | X | X | X | X |
| NYHA Class | X |  | X | X | X | X |
| QoL Questionnaire^7^ | X |  |  | X | X |  |
| Serious Adverse Events |  | X | X | X | X | X |
| Device deficiencies |  | X | X | X | X | X |
| Accessories deficiencies |  | X |  |  |  |  |

^1^ Anti-coagulant and/or anti-platelet medication.

^2^Blood evaluations will include WBC, RBC, hemoglobin, hematocrit, platelets, serum LDH, haptoglobin, reticulocyte, plasma free hemoglobin, serum creatinine (pre-operatively and at discharge visit only) and INR/quick. Plasma free hemoglobin will be collected only at sites that have the local capabilities of performing the evaluation. Blood tests baseline: ideally be taken on admission, but in any case, not ≥ 7 days prior to procedure.

^3^ Ideally subjects should at least have an office visit at 30 Days, 1Y, 3Y and 5Y and if possible at 7Y and 10Y, and phone calls at 2Y, 4Y, 6Y, 8Y and 9Y. If one of the office visits is omitted, every effort should be made to conduct the following visit as office visit.

^4^ Ideally pain score assessment should be collected and entered at post-op day 1, day 3, discharge and 30 days post-operative

^5^ Including collection of images for analysis by Core Lab 3D TEE or 3D TTE images in accordance to standard of care

^6^ CT is optional - only when done as standard of care

^7^ KCCQ-12 questionnaire for all the subjects

# S4_MEMO 4D ECHOCARDIOGRAPHIC PROTOCOL

**ECHOCARDIOGRAPHIC EXAMINATION**

**General Principles**

The echocardiographic examinations should be performed paying attention to patient’s needs. It may be unnecessary to underline the fact that patient care and comfort come first.

In addition, patient cooperation is a prerequisite to obtaining high quality transthoracic echocardiographic studies. To reduce intra-patient variability, all examinations should ideally be performed by the same echocardiographer/sonographer and the same echo machine.

**Patient Preparation**

For the TTE examination, the patient should be placed in the left lateral position. Before starting the echo examination, the patient’s blood pressure, weight and height should be obtained and documented.

For TEE examination, the images should be collected before the start of the cardio-pulmonary bypass and just after coming off cardio-pulmonary bypass after the prosthetic annulus has been implanted.

**Echocardiographic Instrumentation**

After positioning electrodes on the patient’s chest, it will be necessary to optimize the electrocardiographic signal to obtain a QRS complex of adequate size that is clearly visualised on the monitor screen. Recordings of Doppler and M-mode tracings should be performed at 50 or 100 mm/s speed to be able to acquire 2-3 cardiac cycles in each still frame.

For transthoracic studies, the use of 2.5 MHz or higher transducers is required. We recommend the use of a transducer with the highest possible frequency to obtain the best image resolution and an adequate resolution to obtain a good definition of endocardial borders.

Tissue harmonic modality is required to optimize the image, especially for assessment of left ventricular volumes.

Dedicated probes will be used to acquire 3D data sets

**Echocardiographic Examination**

Each echo examination should contain the **Patient’s** **Study ID number** and the **date of examination**, as they are reported on the Echo CRF. To comply with HIPPA and other privacy regulations, do not use patient name.

For color Doppler images, optimize the color sector to the minimum size that shows the whole jet area and maintains adequate frame rate. To ensure intra-patient reproducibility of color flow recordings, the same color map should be maintained in serial studies of the same patient. Color gain should be the highest, providing that no motion artefact is produced by cardiac anatomic structures and there is no speckling superimposed on the image. Wall filters should be set at the lowest level at which cardiac structure motion artefacts are eliminated. Zero velocity line should be placed in the mid of the colour spectrum and the Nyquist limit should be above 50 cm/s.

For each 2D echo view, cine-loop should be recorded acquiring **3 cardiac cycles in patients with sinus rhythm and 5 cardiac cycles in patients with atrial fibrillation**. This can be accomplished by setting the loop record to 3 beats or multiple single or double beats of the same view can be recorded. For M-mode and Doppler tracings, still frames containing at least 3 cardiac cycles are required.

Separate 3D data sets will be acquired for the right and left ventricle, the right and the left atrium, and the mitral and/or the tricuspid valve which has been repaired and had the Memo 4D implanted

***Transthoracic echocardiographic examination - views***

The required echocardiographic recordings for transthoracic studies are listed below*:

Parasternal Long Axis (PLAX) View

- 2D image of left ventricle and aortic root
- High parasternal of the aortic root
- Color flow Doppler of mitral and aortic valves

Right Ventricle Inflow (RVIF) View

- 2D image of RV inflow
- Color Doppler of Tricuspid Valve
- Obtain continuous wave (CW) Doppler of tricuspid regurgitation

Parasternal Short Axis (PSAX) View

- 2D image at basal level
- Color Doppler of aortic and tricuspid valves
- Color Doppler and continuous wave (CW) Doppler of tricuspid regurgitation
- Short axis of LV at papillary muscle level
- Short axis view of the apex (LV should be circular not elliptical)

Apical 4 chamber (AP4) view

- 2D view of all 4 chambers (optimized to avoid foreshortening of the atria)
- 2D view of the left ventricle (optimized by minimizing the depth and avoiding long-axis foreshortening) Frame rate between 60 and 80 fps
- 2D RV focused view
- Color Doppler of mitral and tricuspid valves; both Nyquist settings above 50 cm/s and 30-32 cm/s if there is any regurgitation more than trace.
- CW of MR and /or TR jet (if applicable)
- CW Doppler of mitral and/or tricuspid inflow of the valve with the prosthetic annulus

Apical 5 (AP5) chamber view

- CW Doppler through the aortic valve

Apical 2 (AP2) chamber view

- 2D view of apical 2 chamber (optimized to avoid foreshortening of the left atrium)
- 2D view of the left ventricle (optimized by minimizing the depth and avoiding long-axis foreshortening). Frame rate between 60 and 80 fps
- Color Doppler of mitral valve

Apical long-axis (ALAX) view

- 2D image of apical 3 chamber view (optimized to avoid foreshortening of the left atrium)
- 2D view of the left ventricle (optimized by minimizing the depth and avoiding long-axis foreshortening). Frame rate between 60 and 80 fps
- Color Doppler of mitral valve; both Nyquist settings above 50 cm/s and 30-32 cm/s if there is any regurgitation more than trace
- PW Doppler of LVOT
- CW Doppler through aortic valve

Subcostal View

- 2D image of Subcostal 4 chamber view
- Color Doppler of interatrial septum
- 2D image of inferior vena cava in long axis during free respiration or sniff if there is no vena cava collapse during spontaneous respiration

**3D echocardiography data sets**

**Left ventricle:** the data set should be large enough to include the left ventricular cavity and myocardial wall. The left ventricle should be placed in the center of the data set and depth should be optimized (at its minimum to visualize LV cavity) to improve the temporal resolution. Respiratory maneuvers and/or gain/contrast settings should be optimized in order to visualize the endocardium. The use of the multislice display will help to ensure that the whole left ventricle is included in the data set. The minimal required temporal resolution will be 20 vps.

**Right ventricle**. A dedicated acquisition of the right ventricle should be performed placing the probe more laterally and directing it more towards the right shoulder than is conventionally used to acquire the left ventricle (RV focused apical view). Respiratory maneuvers and/or gain/contrast settings should be optimized in order to visualize the endocardium. The use of the multislice display will help to ensure that the whole right ventricle (and, particularly the anterior wall) is included in the data set. The minimal required temporal resolution will be 20 vps.

**Atria**. Since we expect that atria will be dilated in patients enrolled in this study, we need dedicated data sets for each atria. Each atrium should be placed in the center of the data set and both its depth and volume size should be optimized (at its minimum to visualize the whole atrium) to improve the temporal resolution. Respiratory maneuvers and/or gain/contrast settings should be optimized in order to visualize the endocardium. The use of the multislice display will help to ensure that the whole atrium is included in the data set. The minimal required temporal resolution will be 20 vps.

**Mitral and or tricuspid valve**. Dedicated acquisitions of the mitral and the tricuspid valves will be performed with the valve in the center of the data set and by optimizing the volume size in order to maximize both spatial and temporal resolutions. Respiratory maneuvers and/or gain/contrast settings should be optimized in order to visualize the endocardium. There is no specific acoustic window from which the data sets of the atrio-ventricular valves will be acquired. Use the approach from which the highest quality 2D images can be obtained. Since the main goal of this substudy is to measure the remodeling and the dynamics of the Memo 4D after implantation and its effects on cardiac chamber remodeling, acquisition of high quality 3D data sets is crucial to reach the study goals

The hemodynamic impact of valve repair can be reflected by the transvalvular gradient and also by pulmonary artery pressure. Systolic pulmonary artery pressure will be estimated using CW Doppler interrogation of tricuspid regurgitation jet to calculate right atrio-ventricular gradient. Therefore, care should be taken to record the highest possible tricuspid regurgitant jet velocity.

***Transesophageal echocardiography study - views*** (3 cardiac cycles in sinus rhythm, 5 cardiac cycles in atrial fibrillation)

**4-chamber view** without and with color

**Bicommissural view** without and with color

**Apical long-axis view** without and with color

**RV focused 4-chamber view** to image the tricuspid valve (deep esophageal view) without and with color

**Bicaval view** with visualization of the tricuspid valve

**3D data set of the mitral valve** without and with color

**3D data set of the tricuspid valve** without and with color

**ECHO EXAM AND CASE REPORT FORM**

The echo exam should be labeled using the patient ID, exam date and study interval and should be uploaded in the EDC (IBM) anonymized.

# S5_Table S5_ Site reported echocardiographic findings

|  | **Baseline** | **Discharge** | **30 days** |
| --- | --- | --- | --- |
| **Left Ventricular Ejection Fraction [%]** | | | |
| n | 155 | 159 | 131 |
| Mean(SD) | 61.70 (7.56) | 53.82 (8.22) | 54.79 (7.38) |
| Median | 61.00 | 55.00 | 55.00 |
| IQR | 58.00; 67.00 | 48.00; 60.00 | 51.00; 60.00 |
| **Left Ventricular End-Diastolic Diameter [mm]** | | | |
| n | 114 | 105 | 102 |
| Mean(SD) | 55.27 (14.98) | 52.45 (12.47) | 49.75 (6.57) |
| Median | 55.00 | 52.00 | 50.00 |
| IQR | 50.00; 59.00 | 47.00; 56.50 | 45.00; 54.00 |
| **Left Ventricular End-Systolic Diameter [mm]** | | | |
| n | 61 | 44 | 41 |
| Mean(SD) | 38.31 (10.37) | 39.93 (8.85) | 37.49 (7.12) |
| Median | 38.00 | 39.00 | 37.00 |
| IQR | 33.00; 44.00 | 33.80; 46.00 | 32.00; 41.00 |
| **Left Ventricular End-Diastolic Volume [ml]** | | | |
| n | 88 | 80 | 60 |
| Mean(SD) | 137.81 (47.58) | 115.52 (40.51) | 111.06 (36.89) |
| Median | 140.00 | 110.50 | 111.00 |
| IQR | 100.00; 166.25 | 85.00; 145.95 | 83.80; 131.50 |
| **Left Ventricular End-Systolic Volume [ml]** | | | |
| n | 67 | 65 | 56 |
| Mean(SD) | 54.27 (26.61) | 56.66 (29.16) | 54.24 (25.98) |
| Median | 49.70 | 56.80 | 48.00 |
| IQR | 35.00; 64.00 | 35.30; 71.60 | 37.50; 65.15 |
| **Left Atrial Volume [ml]** | | | |
| n | 83 | 42 | 50 |
| Mean(SD) | 92.23 (40.34) | 92.79 (39.43) | 85.95 (36.54) |
| Median | 85.87 | 91.00 | 84.17 |
| IQR | 62.00; 120.00 | 62.00; 120.00 | 55.90; 110.00 |
| **Mean Mitral Pressure Gradient [mmHg]** | | | |
| n |  | 140 | 113 |
| Mean(SD) |  | 3.55 (1.47) | 3.17 (1.63) |
| Median |  | 3.00 | 3.00 |
| IQR |  | 2.43; 4.40 | 2.00; 3.75 |
| **Systolic Anterior Motion, N (%)** |  | N available = 123 | N available = 109 |
| Yes | NA | 1 (0.8%) | 2 (1.8%) |
| No | NA | 122 (99.2%) | 107 (98.2%) |
| **Mitral Regurgitation, N (%)** | N available = 153 | N available = 160 | N available = 137 |
| None | 1 (0.7%) | 88 (55.0%) | 62 (45.3%) |
| Trace | 0 | 53 (33.1%) | 55 (40.1%) |
| Mild | 1 (0.7%) | 17 (10.6%) | 16 (11.7%) |
| Moderate | 9 (5.9%) | 2 (1.3%) | 2 (1.5%) |
| Severe | 142 (92.8%) | 0 | 2 (1.5%) |

**SD: standard deviation, IQR: interquartile range**

# S6_Approval numbers from Institutional Review Boards or Ethic Committees

- Kantonale Ethikkommision Zürich; Approval number: 2021-D0045
- Comitato Etico Regionale delle Marche; Approval number: 2021 463
- Comité de protection des personnes Ile de France I; Approval Number: 21.03296.055912-MS01.1
- Comitato Etico di Brescia; Approval Number: NP 4743
- Ethikkommission der Medizinischen Universität Innsbruck; Approval number: 1073/2022
- Comitato Etico dell’Ospedale San Raffaele; Approval number: 399/20221
- Ethik-Kommission bei der Landesärztekammer Hessen; Approval number: 2021-2756-zvBO
- Comitato Etico ASL Lecce PugliaSalute; Approval number 04008300750: see previouse email
- ADVARRA
- Ethink-Kommissiond der FAU; Approval number: 95_21 B
- Comité de protection des personnes Ile de France I; Approval number: 21.03296.055912-MS02
- Comitato Etico di Area Vasta Emilia Centro; Approval number: 341/2022
- Comitato Etico Palermo 1; Approval number 06/2021
- Yorkshire & The Humber - Sheffield Research Ethics Committee; Approval number 304706
- Comitato Etico Unico Regionale del Friuli-Venezia Giulia

1. Stone GW, et al. Clinical trial design principles and endpoint definitions for transcatheter mitral valve repair and replacement: part 2: endpoint definitions: A consensus document from the Mitral Valve Academic Research Consortium. Eur Heart J. 2015 Aug 1;36(29):1878-91. doi: 10.1093/eurheartj/ehv333. Epub 2015 Jul 13. [↑](#footnote-ref-2)
2. Lancellotti, P et al. Recommendations for the imaging assessment of prosthetic heart valves: a report from the European Association of Cardiovascular Imaging endorsed by the Chinese Society of Echocardiography, the Inter-American Society of Echocardiography, ... Eur Heart J Cardiovasc Imaging, 17(6):589-90. [↑](#footnote-ref-3)
3. Spertus, J. A., et al.. Interpreting the Kansas City Cardiomyopathy Questionnaire in Clinical Trials and Clinical Care: JACC State-of-the-Art Review. J Am Coll Cardiol, 76(20):2379-2390. [↑](#footnote-ref-4)
4. Ryomoto, M et al (2014). Is physiologic annular dynamics preserved after mitral valve repair with rigid or semirigid ring? Ann Thorac Surg, 97(2):492-7. [↑](#footnote-ref-5)
5. Ryomoto, M et al (2017). Physiological mitral annular dynamics preserved after ring annuloplasty in mid-term period. Gen Thorac Cardiovasc Surg, 65:627–632. [↑](#footnote-ref-6)
6. Gorman, J. H et al. (2006). Pathophysiology of ischemic mitral insufficiency: does repair make a difference? Heart Fail Rev, 11(3):219-29. [↑](#footnote-ref-7)
7. Kaplan, S. R., et al. (2000). Three-dimensional echocardiographic assessment of annular shape changes in the normal and regurgitant mitral valve. Am Heart J, 139(3):378-87. [↑](#footnote-ref-8)
8. van Wijngaarden, S. E, et al (2018). Three-dimensional assessment of mitral valve annulus dynamics and impact on quantification of mitral regurgitation. Eur Heart J Cardiovasc Imaging, 19(2):176-184. [↑](#footnote-ref-9)
